# Analyzing engram reactivation and long-range connectivity

**DOI:** 10.1016/j.xpro.2024.102840

**Published:** 2024-01-26

**Authors:** Ron Refaeli, Tirzah Kreisel, Inbal Goshen

**Affiliations:** 1 Edmond and Lily Safra Center for Brain Sciences (ELSC), The Hebrew University of Jerusalem, Jerusalem 91904, Israel

**Keywords:** Microscopy, Model Organisms, Neuroscience, Behavior

## Abstract

Here, we present a protocol for marking engram cells to efficiently measure reactivation levels and their projection pathways. We describe steps for genetic manipulation utilizing transgenic mice and viral infections, labeling engram cells, and a modified version of CLARITY, a tissue-clearing technique. This protocol can be adapted to various research inquiries that involve assessing the overlap of cell populations and uncovering novel long-range connectivity pathways.

For complete details on the use and execution of this protocol, please refer to Refaeli et al. (2023).^1^

## Highlights

• Tagging two populations of engram cells in the same mouse
• Measuring the level of engram reactivation using transgenic mice and viral vectors
• Tracing axonal paths of engram cells in cleared brains
• Using a modified CLARITY tissue-clearing technique

## Abstract (editor highlights)

Publisher’s note: Undertaking any experimental protocol requires adherence to local institutional guidelines for laboratory safety and ethics.

## Graphical abstract

**Figure:**
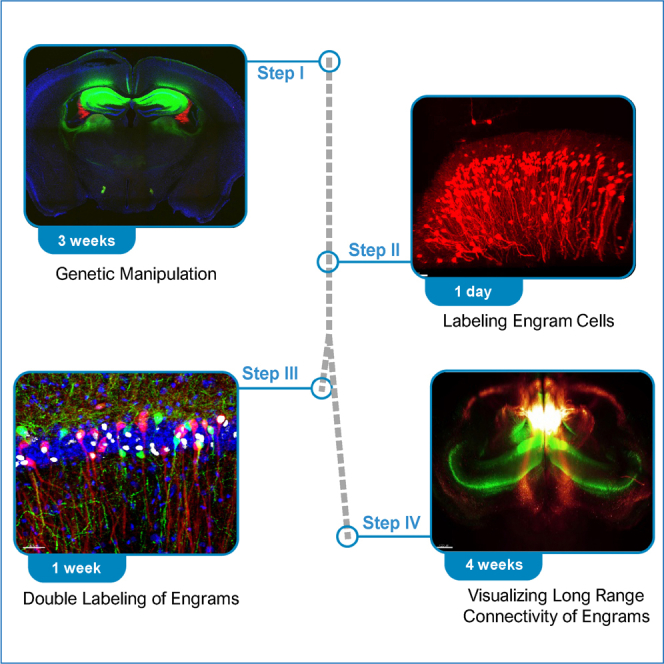

## Before you begin

Before initiating the following protocol, it is essential that the lab gains access to specific requirements, including genetic manipulation, immunostaining, tissue clearing, and commercialized software. Here are the key elements needed.

1. *Genetic manipulation*: Involves using TG mice (for example, Ai14 reporter mice^2^) and viral vectors (for example, AAV5-c-Fos::cre^ER^), as described in the protocol.
2. *Immunostaining:* Primary antibody (rabbit anti c-Fos, Synaptic System #226008) and secondary antibody (donkey anti rabbit, AF 647 #711-605-152, Jackson laboratory).
3. The CLARITY tissue clearing technique requires multiple premade ingredients. Carefully read the protocol and tables appended to it before you begin. Details can be found in the following papers.^1^^,^^3^

### Institutional permissions

Any experiments on live vertebrates or higher invertebrates must be performed in accordance with relevant institutional and national guidelines and regulations. In this protocol example, all experimental protocols were approved by the Hebrew University Animal Care and Use Committee and met the guidelines of the National Institute of Health guide for the Care and Use of Laboratory Animals.

If missing, your experimental procedure will need to acquire ethical permissions from your relevant institutions prior to this protocol execution.

## Key resources table

**Table:** 

REAGENT or RESOURCE	SOURCE	IDENTIFIER
**Antibodies**		

Rabbit anti-c-Fos (1:10,000)	Synaptic Systems	#226 008
Alexa Fluor 647 donkey anti-rabbit (1:500)	Jackson Laboratory	#711-605-152

**Bacterial, plasmids, and virus strains**		

pFosCreER	Addgene	#46388
AAV5-c-Fos::cre^ER^	ELSC Vector Core Facility	N/A
AAVretro-CaMKII::iCre	ELSC Vector Core Facility	N/A
AAVretro-CaMKII::GFP	ELSC Vector Core Facility	N/A
pAAV-CaMKII-eGFP	Addgene	#50469
pCAG-Flex-RG	Addgene	#48333

**Chemicals, peptides, and recombinant proteins**		

DAPI	Sigma	D9542
PBS	Sartorius	020235A
Glycerol anhydrous	Biolab	071205
Ethylene glycol	Sigma	324558
Acrylamide	Bio-Rad	#161-0140
Bisacrylamide	Bio-Rad	#161-0142
VA-044 initiator	Wako	011-19365
Boric acid	Sigma	#B7901
Tris base	Bio-Lab	002009239100
Sodium dodecyl sulfate (SDS)	Sigma	#L3771
Triton X-100	ChemCruz	#sc-29112A
CLARITY specific RapiClear	SunJin Lab	#RCCS002
Clozapine *N*-oxide (CNO)	Tocris Bioscience	#4936
4-Hydroxytamoxifen (4-OHT)	Sigma	H7904
Sunflower oil	Sigma	S5007
Absolute ethanol	Merck	64175
Bovine serum albumin	MP Biomedicals	9048-46-8
Mounting medium	Dako	S3025
Coverslip	Marienfeld	0101242
Hot glue	Amazon	N/A

**Deposited data**		

Mendeley Data:https://data.mendeley.com/datasets/9x9xph4v46/1		

**Experimental models: Organisms/strains**		

B6;129S6-Gt(ROSA)26Sor^tm14(CAG-tdTomato)Hze^ (Ai14)	Jackson Laboratory	J-Stock No: 007908

**Software and algorithms**		

EthoVision tracking software version 13	Noldus	
Olympus Fluoview Viewer version 4.2	Olympus	
Bitplane Imaris 9.1.2	Bitplane	
syGlass	IstoVisio	
MATLAB 2018	MathWorks	
IBM SPSS statistics	IBM Analytics	

## Materials and equipment

**Table:** For Engram tagging using 4-OHT

Reagent	Final concentration	Amount
4 hydroxytamoxifen (4-OHT)	0.5%	3 mg
Absolute Ethanol	20%	120 μL
Sunflower oil (or any kind of non-toxic oil)	80%	480 μL
Total		600 μL

For an Ai14 mouse weighting 30 g, inject 150 μL of the 3 mg 4-OHT solution for a total injection of 25 mg/kg.

First, weight the 4-OHT under chemical hood to prevent inhalation. Dissolve the 4-OHT in Ethanol. This procedure requires continuous mixing for several minutes to allow sufficient dissolving. Only after the 4-OHT dissolves, add the Sunflower oil. The oil and the ethanol will create two phases, so make sure to remix (by manually shaking the tube) just before injection. Evaporation of the Ethanol is not necessary for these volumes in this protocol.

For CLARITY protocol, prepare the following solutions.

**Table:** Prepare a Hydrogel solution for fixation of the brain tissue prior to the clearing process

Hydrogel solution (1 L)		
Reagent	Final concentration	Amount
VA-044 Initiator	2.5%	2.5 g
4% PFA (in PBS)	3.6%	900 mL
40% Acrylamide	2%	50 mL
2% Bisacrylamide	0.1%	50 mL
Total		1 L

Prepare the Hydrogel solution on ice, to prevent premature polymerization of the ingredients. Prepare the solution under a chemical hood to avoid inhalation or skin contact with the toxic ingredients.

Clearing solution removes the lipids from the tissue resulting in transparent tissue. Keep the tissue in the solution until the tissue is transparent. Make sure not to keep it in the clearing solution for too long, as the rigidity of the tissue will critically diminish. Depending on the thickness of the tissue, it should be extracted from the clearing solution after 2–4 weeks.

**Table:** 

Clearing solution (1 L)				
Reagent	Clearing solution 1		Clearing solution 2	
	Final concentration	Amount	Final concentration	Amount
Boric acid (M.W. = 61.83 g/mol)	200 mM	12.366 g	55 mM	3.4 g
Sodium Dodecyl Sulfate (SDS)	8%	80 g		80 g
Tris base (M.W. = 121.14 g/mol)	N/A	0 g	100 mM	12.1 g
Distilled water (DW)	Fill to 1 L		Fill to 1 L	
NaOH	Titration to reach pH of 8.5		No need for titration	

Clearing Solution 1 does not include Tris base, since it may react with PFA residue. Any solution containing boric acid and SDS should be handled under a fume hood to avoid inhalation or skin contact.

• Phosphate-buffered Saline with 0.5% Triton X-100 (0.5% PBST): add 5 mL Triton X-100 in 1 L PBS.

**Table:** For cFos protein staining, prepare the following solutions:

Cryoprotectant		
Reagent	Final concentration	Amount
Glycerol anhydrous	25%	250 mL
Ethylene Glycol	30%	300 mL
10× PBS	10%	100 mL
DW	35%	350 mL
Total		1 L

**Table:** 

Blocking solution		
Reagent	Final Concentration	Amount
Bovine serum Albumin	1%	0.5 g
10% Triton X-100 in PBS	0.3%	1.5 mL
PBS	N/A	Fill to 50 mL

• Primary antibody solution: 1:10,000 Rabbit anti c-Fos in Blocking solution.

**Table:** 

Secondary antibody		
Reagent	Final Concentration	Amount
Bovine serum Albumin	1%	0.1 g
PBS	N/A	10 mL
AF 647, Donkey anti Rabbit	1:500	20 μL

• DAPI staining: Stock of 1:50 DAPI in DDW. 1:1,000 in PBS for final concentration of 1:50,000 for 5–10 min.

## Step-by-step method details

### Stereotaxic virus infusion

**Timing: 3–5 weeks**

This procedure details how to infect mouse brain cell with AAV-virus.

1. Expression of a red fluorophore protein in cFos expressing cells at an Ai14 mouse.
  a. Using a brain atlas, find the relevant coordination.
  b. Inject the viral vector (AAV5-cFos:: Cre^ER^) into the chosen brain structure of Ai14 mice.^2^ For example, in this protocol, 400 nL was injected into the dorsal hippocampus (AP -1.85 mm, LM ±1.4 mm, DV -1.5 mm relative to Bregma). Note: All AAV viruses in this protocol are defined as BSL1.
2. Let the viral vector infect the cells at the injected site.

***Note:*** Three weeks are sufficient for nonfunctional vectors such as in this protocol example, but functional vectors such as GCaMP, DREDDs and optogenetic tools require a longer infection period (>4 weeks).

### Labeling engram cells in-vivo

**Timing: 1 h**

The following steps detail how to do label activated cells after the behavioral paradigm.

3. Perform the behavioral paradigm. In this protocol, the behavioral paradigm^2^ is the recent recall of contextual fear conditioning.

***Note:*** the next step follows after only 1 h, and the timing is crucial.

4. Using a 23 gauge needle to intraperitoneally (IP) inject 150 μL 4-OHT to a mouse weighting 30 g (25 mg/kg)1 h after the behavioral paradigm.^4^

**CRITICAL:** The 4-OHT solution has to be freshly prepared and cannot be stored.

***Note:*** 4-OHT level in the blood stream peaks after 1.5 h and stays high for several hours.^4^ For that reason, some protocols may suggest a variety of time intervals between the IP injection and the behavioral performance (from 1 h before to 3 h later). All are valid as long as the time interval set for your protocol remains the same for all mice. Mice and cages should be treated after 4-OHT injection according to your animal facility guide lines.

5. Let the fluorophore express.

***Note:*** Two days are sufficient for somatic expression, but >4 weeks are recommended for axon tracing.

*At this point the protocol splits into two paths – double labeling or whole brain imaging.* For clearing tissue protocol, jump to step 29.

### Labeling of 2^nd^ engram cells

**Timing: 3 days**

This section describes how to prepare the brains for immunohistochemistry.

6. Perform the second behavioral paradigm. In this protocol, the second behavioral paradigm is remote recall of contextual fear conditioning 26 days after the previous recall (Figure 1).

**Figure 1:**
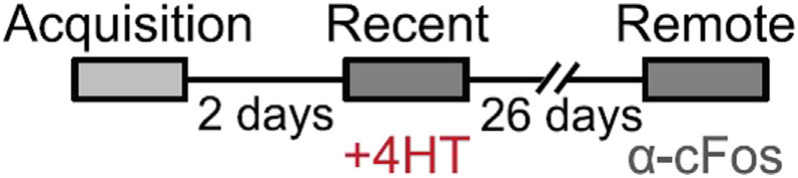
Scheme of the behavioral paradigm used in this protocol Mice were trained in Contextual fear conditioning and recall this aversive memory after two days and 28 days. Both recall events were followed by engram group tagging, the first in-vivo, and the second by IHC. Any other behavioral paradigm with two time points can be performed similarly.
7. 90 min after the behavioral paradigm is over, sacrifice (by perfusion) the animal, remove the brain, and immerse it in 4% PFA for immediate fixation. Keep the brain in PFA overnight in 4°C.
8. Under a chemical hood, move the brain from PFA to a 30% Sucrose 4°C in PBS (w/v) for dehydration until it sinks, indicating equilibrium. This usually takes ∼48 h.
9. Slice the brain into thin sections containing your relevant brain structure. In this protocol, we sliced the tissue into 50 μm sections using a freezing microtome for sectioning, targeting the hippocampus and the ACC.
10. Store the slices in Cryoprotectant and keep at 4°C. Cryoprotectant can be store indefinitely in RT. Each set of sample requires approximately 500 μL

### Immunohistochemistry (IHC, staining for c-Fos expressing cells)

**Timing: 1 week**

The following steps detail how to preform staining for activated cells using the c-Fos antibody.

11. Move the slices from Cryoprotectant to PBS for 15 min on shaker. Replace the PBS twice (15 min each) to make sure all Cryoprotectant remnant is removed.
12. Move the free floating slices to a blocking solution with mild shaking (70 rpm) for 1 h at room temperature (∼25°C). Meanwhile, prepare the primary antibody solution.
13. Move the slices to the primary antibody solution. In this protocol, we stain against the c-Fos protein, which requires mild shaking at 4°C for 7 days.

***Note:*** based on comprehensive examinations, 7 days are needed for a reliable staining with minimal background noise.

14. Move the slices from the primary antibody into the PBS. Let it shake for 15 min and move to PBS for the second time (15 min on shaker).
15. Move the slices into the secondary antibody solution. In this protocol we use AF 647, to visualize all stained proteins in far-red fluorophore, which require 2 h shaking (70 rpm) in room temperature.
16. Move slices to PBS for 15 min.
17. Move slices into DAPI (1:50,000) for 5 min to stain all cell nuclei.
18. Mount slices on slides.
19. Let the slices dry on the slide for 20 min and apply 1–2 drops of mounting medium before sealing with a coverslip.

### Imaging (confocal microscope)

**Timing: 15 min per image**

After staining brains are imaged using a confocal microscope for further analysis.

20. Load the relevant slices onto your microscope. In this protocol, we used a confocal microscope to separate between the imaged planes.
21. Make sure you image all of your colors of interest. In this protocol, we imaged blue (DAPI), red (cells tagged at 1^st^ time point), and far-red (cells tagged at 2^nd^ time point).

***Note:*** Parameters of each image should remain the same between different images (i.e. objective, magnification, depth size, laser intensity etc.)

### Cells localization and analyzing (imaris)

**Timing: 1 day**

This section details how to use the Imaris software to count different colored cells, and analyze the overlap between them.

**Figure 2:**
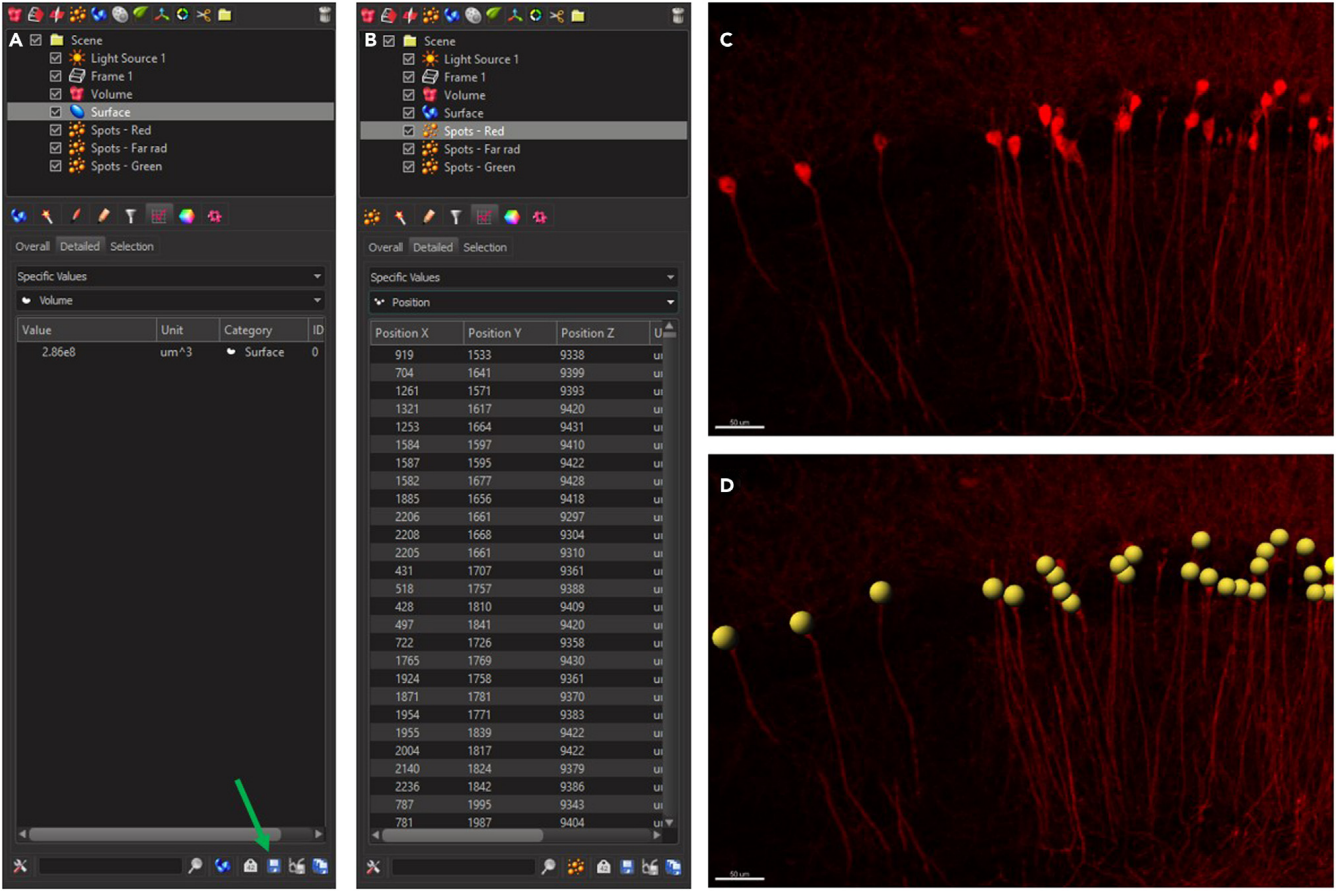
Image analysis using Imaris (A) The ‘Surface’ option on Imaris is used to define boundaries. The green arrow indicates the option to download the desired excel data. (B) The ‘Spots’ option used to define somata position. (C) TdTomato+ cells in the pyramidal layer of the CA1. (D) Marking the somata of all red cells using the Spots option.

***Note:*** Other software (commercial or open source) are available. If using other software, this specific step of “cells localization” should be modified to fit your software. It does not affect any previous or following steps.

22. In this protocol, we use Imaris software to open and analyze the image (Figure 2).

23. Using the ‘Surface’ option on Imaris, define the boundaries of your brain region.
24. Export the volume of your surface via Excel.
25. For each channel, mark all somata locations inside the surface by using ‘Spots’ option.
26. Export the XYZ positions of all spots from each color channel via Matlab, Python or Excel.
27. Count the number of cells expressing both colors, by manual counting or via the code on the next page, or your own code.

***Note:*** using code eliminates possible researcher biases.

28. Using the following equation, define if the different colored cells overlap less, more, or as expected by random tagging:
  $$\frac{\frac{amount\;of\;counted\;x\;and\;y}{amount\;of\;counted\;x}}{\frac{\frac{amount\;of\;counted\;x}{DAPI}\times \frac{amount\;of\;counted\;y}{DAPI}\times DAPI}{amount\;of\;counted\;x}}$$
  where 'x' represents one color and ‘y’ represents the second (i.e., 'how many of x cells were also y cells, out of the total amount of x cells').
  a. A simplified Matlab code is attached below for basic guide-lines on how to define the overlap of different colored cells:

% Go do directory containing 'red' (1st time point), 'FR' (2nd time point),

% 'green' (CA1->ACC), 'surface' (outlines of the CA1), 'surface_min_max', (borders of the surface), excel files containing Surface data.

allMat = dir('∗.mat');

allCSV = dir('∗.csv');

distance_threshold = 5; % define what is the maximal distance between two spots to represent the same cell. Notice the difference between pixel size and real size.

% Two options available: count how many DAPI cells you have in each slice,

% or calculate the average density of DAPI cells, as indicated below:

meanDAPI = ; % average of cells per volume (mean_vol)

mean_vol = ; % average size of CA1 volume, to calculate amount of cells.

j=1;

for i=1:length(allMat)

 if strfind(allMat(i).name,'FR')

  FR = load(allMat(i).name);

  FR = struct2cell(FR);

  FR = FR{1};

  FR = FR(:,1:2);

% for FR (far red, 2ND engram group) loading the matrix of spots position.

 elseif strfind(allMat(i).name,'red')

  red = load(allMat(i).name);

  red = struct2cell(red);

  red = red{1};

  red = red(:,1:2);

% for red (1st engram group) loading the matrix of spots position.

 elseif strfind(allMat(i).name,'green')

  green = load(allMat(i).name);

  green = struct2cell(green);

  green = green{1};

  green = green(:,1:2);

% for green (CA1->ACC) loading the matrix of spots position.

 elseif strfind(allMat(i).name,'min_max')

% For each image, 'surface_min_max' is chronically the last item, meaning all other parameters are for the relevant slice investigated.

  red2FR = pdist2(FR,red); % given "red", how many are also "FR"?

  min_red2FR = min(red2FR);

  min_red2FR_conditioned = min_red2FR < distance_threshold;

  FCFRandRED(j) = sum(min_red2FR_conditioned)/length(red)∗100;

% similar code can be done to calculate overlap between ACC projecting cells (green) and 1st engram cells (red), etc.

  for p = 1:length(allCSV);

   if strncmpi(allMat(i).name,allCSV(p).name,4)

    csvdata = xlsread(allCSV(p).name);

    this_volume = csvdata(1); % from the CSV file, extracting the volume size (downloaded from the imaris)

    relative_size = this_volume/mean_vol;

    expectedDAPI = meanDAPI∗relative_size; % number of DAPI cells in this volume.

   end

  end

  PERCENTAGE_RED(j) = (length(red)/expectedDAPI)∗100; % percentage of 1st engram cells

% Same calculation can be done on the other colors percentage of total amount of cells

FRandREDbyDAPIrelative2red(j)=((((length(FR)/expectedDAPI)∗(length(red)/expectedDAPI))∗expectedDAPI)/length(red))∗100;

% Expected overlap between recent and remote engram cells

% Same calculation can be done on the overlap between different colors.

  % folding the results relative to expected

  FoldOfRedAndFRdividedByExpected(j) = FCFRandRED(j)/FRandREDbyDAPIrelative2red(j); % two time points overlap

% Same calculation can be done on the ratio between other sub groups of cells populations (such as 1st engram cells and CA1->ACC projecting cells)

  j=j+1;

 end

end

### Tissue harvesting and fixation

**Timing: 3 days**

The first stages if brain clearing involves a good removal of the blood from the brain and then fixation.

29. >4 weeks after the 4-OHT injections (part 2), perfuse the animal first with PBS and then with 4% PFA. Make sure to remove all blood remnants.

***Note:*** Removal of any blood residue is essential, since the blood cells are extremely auto fluorescent, which weakens the ability to correctly analyze the data extracted from a thick tissue such as the brain. While performing the perfusion, you can assess the removal of blood by observing the whitening of internal tissues such as the liver.

30. Leave the brain in 4% PFA overnight at 4°C.
31. Replace PFA with Hydrogel solution and keep it for 2 days at 4°C. Make sure the PFA and the Hydrogel solution are cold, to prevent premature cross links in the Hydrogel solution. The Hydrogel solution can be kept at −20°C indefinitely.

***Note:*** Elaborated protocol for the CLARITY procedure can be found in here^3^

### Polymerization

**Timing: 4 h**

The next step in the clearing procedure includes removing all free oxygen from the tube and then removal of the Hydrogel solution.

32. Replace the air in the tube containing the brain with N_2_ to remove all free oxygen (O_2_) which interferes with the polymerization process (Figure 3). Falcon tubes can be linked together in a linear fashion, enabling concurrent gas exchange for multiple tissues.

**Figure 3:**
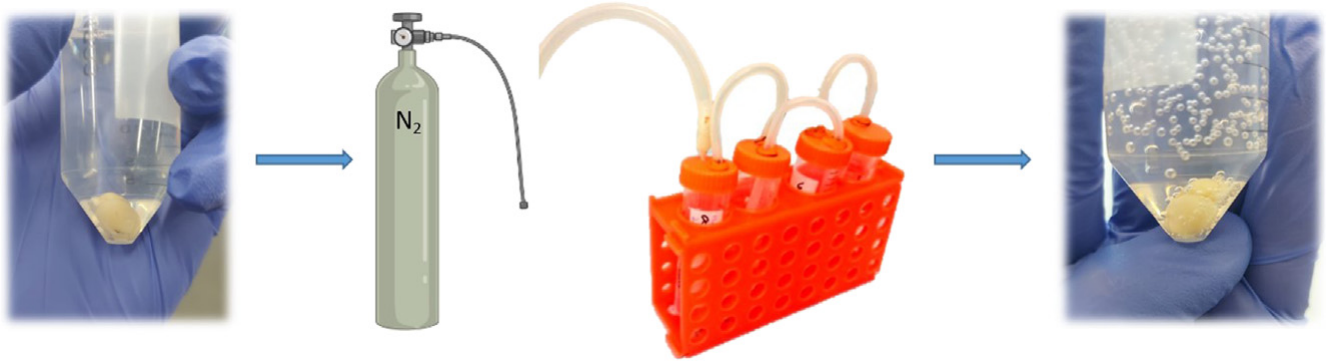
Polymerizing the brain This step involves immersing the fixed tissue in a hydrogel solution and substituting all oxygen with nitrogen. For multiple samples, nitrogen can be replaced with oxygen simultaneously in several tubes (optional). The brain and hydrogel solution around it are then heated to enable polymerization. Over time, the tissue and the surrounding solution combine to form a monomer.
33. Heat the tube to 37°C in a bath for 4 h to let the tissue polymerize.

In a chemical hood, remove the extra hydrogel solution (now in the form of a gel). Using soft-surface tissue such as Kimwipe, make sure to remove all Hydrogel remnant since its ingredients react with the ingredients in the next step (problem 1).

***Note:*** The components of the Hydrogel solution interact with any N-group residue,^5^^,^^6^ predominantly proteins, DNA, and RNA, and excluding lipids

### Lipids removal

**Timing: 1–4 weeks**

The following step include immersing the brains in Clearing solutions 1 and 2 until the tissue is transparent.

34. Move the tissue to a 50 mL tube containing 15 mL of Clearing Solution 1, and leave overnight at 40°C shaker (70 rpm).. Seal the tube with an aluminum cover to prevent light Clearing solution 1 can be stored at RT indefinitely.
35. This solution contains a high percentage of SDS, a detergent to detach lipids
36. In the meantime, create a perforated tube for the next step. Make small holes (10–15 holes, 2–3 mm in diameter) at the bottom of a 50 mL tube to enable the solution flow in and out of the tube. Depending on the sample size, make sure that the holes are large enough to facilitate solution movement but narrow enough to avoid tissue loss.
37. Move the tissue to the perforated tube immersed in a 2 L beaker filled with Clearing Solution 2 on a stirrer hot plate (For more details see^3^). Leave the tissue in this beaker to stir until the tissue becomes transparent.

***Note:*** Two distinct clearing solutions are required due to the presence of Tris base in Clearing Solution 2, which aids in the clearing procedure but could potentially interact with any remaining Hydrogel Solution (problem 1). Therefore, employing Clearing Solution 1 initially helps mitigate the potential for this reaction. Clearing solution 2 can also be stored at RT indefinitely.

### Detergent removal before complete transparency

**Timing: 5 days**

The following steps include several solution replacements to assure complete detergent removal.

38. Move the clear tissue to 0.5% PBST (triton) for 24 h at 37°C with mild shaking in a shaker incubator.
39. Replace the solution with new 0.5% PBST for another 24 h at 37°C with mild shaking.
40. Replace the solution with new 0.5% PBST for another 24 h at room temperature with mild shaking.
41. Replace the solution with PBS for another 24 h at room temperature with mild shaking.
42. Replace the solution with new PBS for another 24 h at room temperature with mild shaking.

### Complete transparency

**Timing: 1–2 days**

This is the final stage in acquiring a clear tissue.

43. Move the tissue into >5 mL CLARITY Specific RapiClear (CSRC). Make sure to dry off as much of the PBS as possible, to prevent a change in the CSRC liquid ingredient ratio and consequently its refractive index. Keep it at 37°C overnight with mild shaking, sealed with aluminum.
44. Move the tube to a shaker at room temperature, and shake until it reaches full equilibrium, i.e., no visible ripples occur near the border of the brain and the solution surrounding it (1–2 days). At this time point the tissue will reach full transparency (problem 2).

### Chamber preparation (for confocal imaging)

**Timing: 30 min**

In order to image the brain under a microscope, an appropriate chamber for the sample needs to be obtained. Here we describe a simple way of making a chamber.

***Note:*** this chamber works for upright confocal or 2-photon microscope setup. If imaging under light sheet microscope, several changes should be applied to the chamber preparation.

45. Place the sample on a slide. Make sure to leave enough space between the tissue and the edges of the slide.
46. Using a hot glue gun, create walls almost as high as the tissue. Make sure to leave a 4–5 mm gap, not sealing the walls entirely (Figures 4A and 4B). This gap will be sealed in sub-step 14.

**Figure 4:**
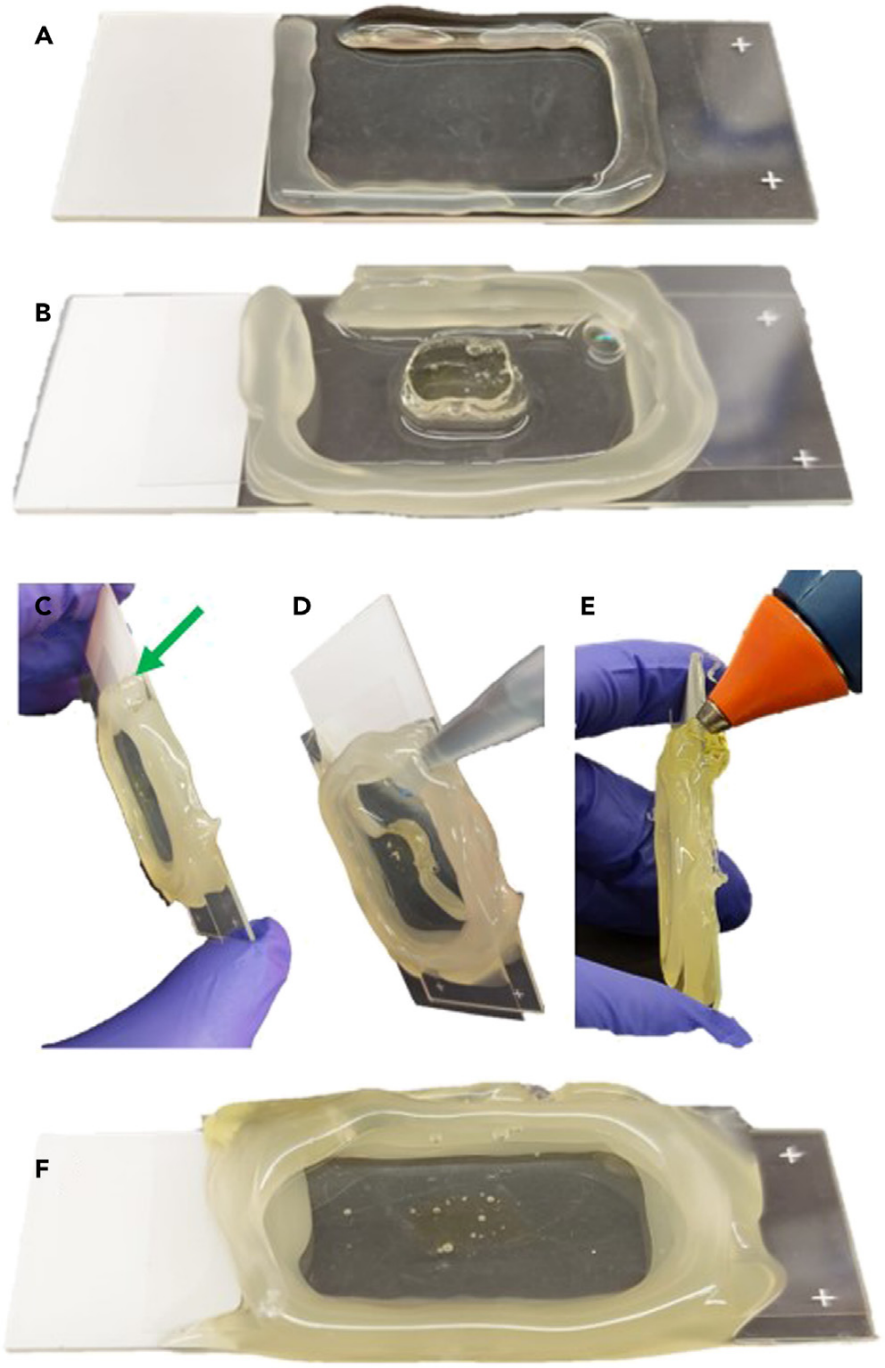
Chamber preparation (A) Preparing hot glue walls ensuring ample space for the tissue and leaving a small gap (upper left corner). (B) Constructing the walls to match the height of the tissue. After sealing the chamber with a coverslip (C), it can be easily filled with CSRC (D). (E) Closing the gap through which the CSRC was filled. (F) Once the chamber is sealed, an additional layer of hot glue is added above the coverslip to contain the immersion liquid. The tissue exhibits sufficient transparency for imaging under the microscope.
47. Apply 1–2 drops of the CSRC on top of the tissue to prevent the formation of bubbles in the next steps.
48. Apply the last level of the walls, making them as high as the tissue.
49. Before the hot glue dries, seal the top of the slide with coverslip. Make sure to place it as evenly as possible, ensuring that no holes between the still liquid glue and the coverslip are formed.
  a. If the objective you will use in the microscope is immersed, apply another layer of hot glue above the coverslip. It will allow better containment of the immersion liquid and assist with preventing holes from forming between the hot glue walls and the coverslip.
50. Via the gap left between the walls, fill the chamber with CSRC (Figures 4C and 4D).
51. Seal the gap with hot glue. Make sure not to leave any bubbles (Figures 4E and 4F).

### Imaging (Olympus confocal microscope)

**Timing: 10–50 h**

Here we present several points needed to be considered when imaging the cleared tissue under the microscope.

52. When imaging thick tissue, several parameters should be taken into account:
  a. The working distance (W.D.) of the objective should align with the intended imaging goal. Entire brain requires at least 5 mm W.D.
  b. Magnification is chosen to suit the specific inquiry at hand, such as locating somata, dendrites, or axons.
  c. Imaging will take several hours or even days. Make sure your facility is suitable for such demands.
  d. When using an immersed objective for several days, the immersed solution needs to be refilled every few hours. Water, for example, needs to be replete every few hours. If evaporated entirely, the image will turn dark.

### Counting cells or axons (SyGlass or imaris)

**Timing: 1 day**

This is the final step, presenting an example of an analysis from the cleared brain image.

53. Open the image in the Imaris software (Figure 5).
  a. Other software (commercial or open source) are available. If using other software, this specific step of counting should be modified to fit your software. It does not affect any of the previous or next steps.

**Figure 5:**
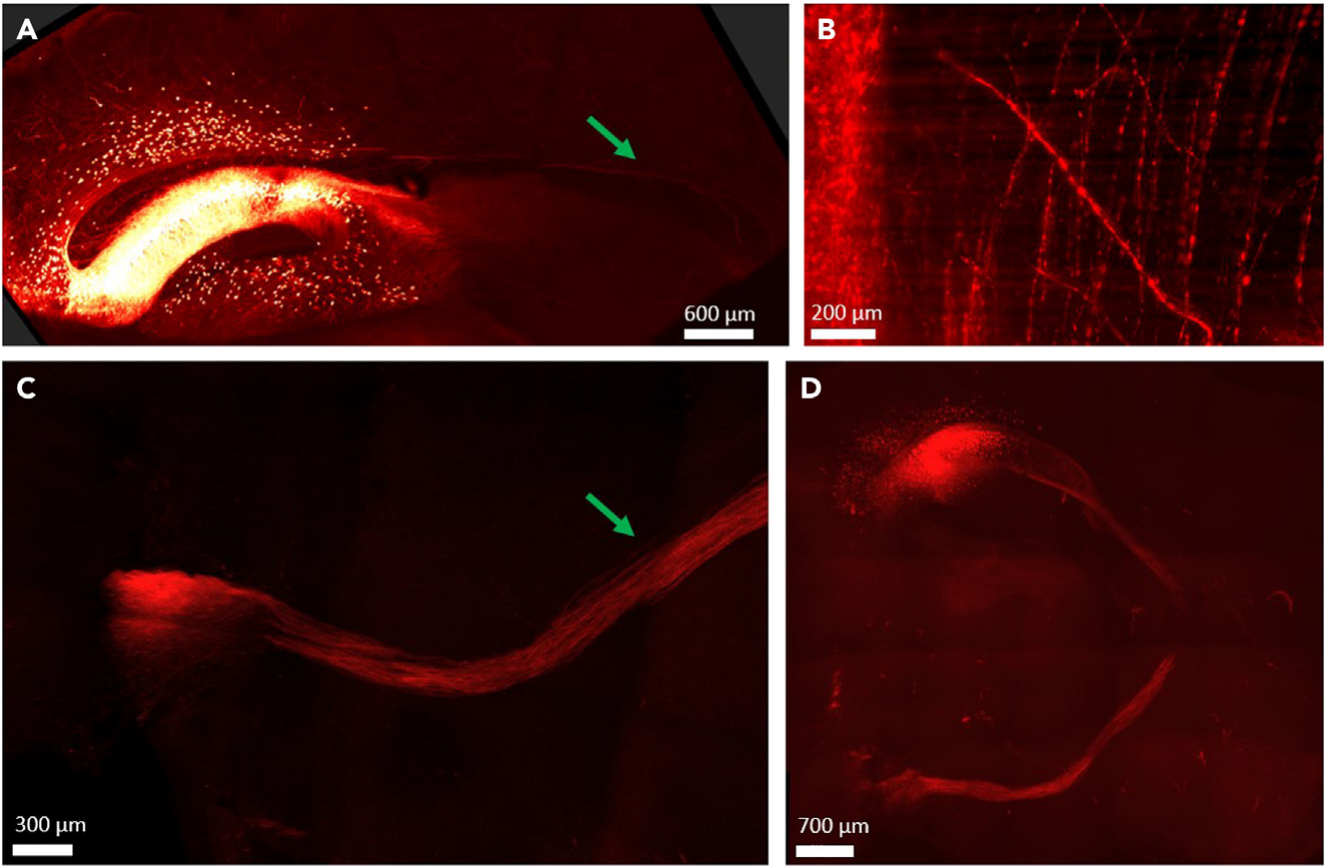
Tracing single neurons and single axons inside an entire clear brain (A) Engram cells at the dorsal CA1 region of the hippocampus of a cleared brain express TdTomato (red). Projections pathways can be easily targeted, heading toward the ACC (arrow). Scale bar = 700 μm. (B) An entire brain was imaged, at a resolution sufficient to separate and count single axons when magnified. Scale bar = 100 μm. (C) Projections can be explored throughout the entire brain. As shown here, this bundle of axons reaches the mammillary bodies (dashed line) via the Fornix (arrow). Scale bar = 300 μm. (D) Using CLARITY allows answering exploratory questions regarding the brain. i.e., not just ‘does the CA1 interact with the ACC?’ but ‘toward which areas does the CA1 sends projections?’ Scale bar = 700 μm.
54. Save the image (as ims file). ims files are compatible with the SyGlass software.
55. Open the ims file in SyGlass. The SyGlass software allows virtual reality exploration of biological data, allowing more accurate manual counting of axons or cells.
  a. Other software can provide the similar results whether using virtual reality (VR) or not. This protocol recommends tracing axons via VR to avoid miscounting of thin projections.

## Expected outcomes

**Figure 6:**
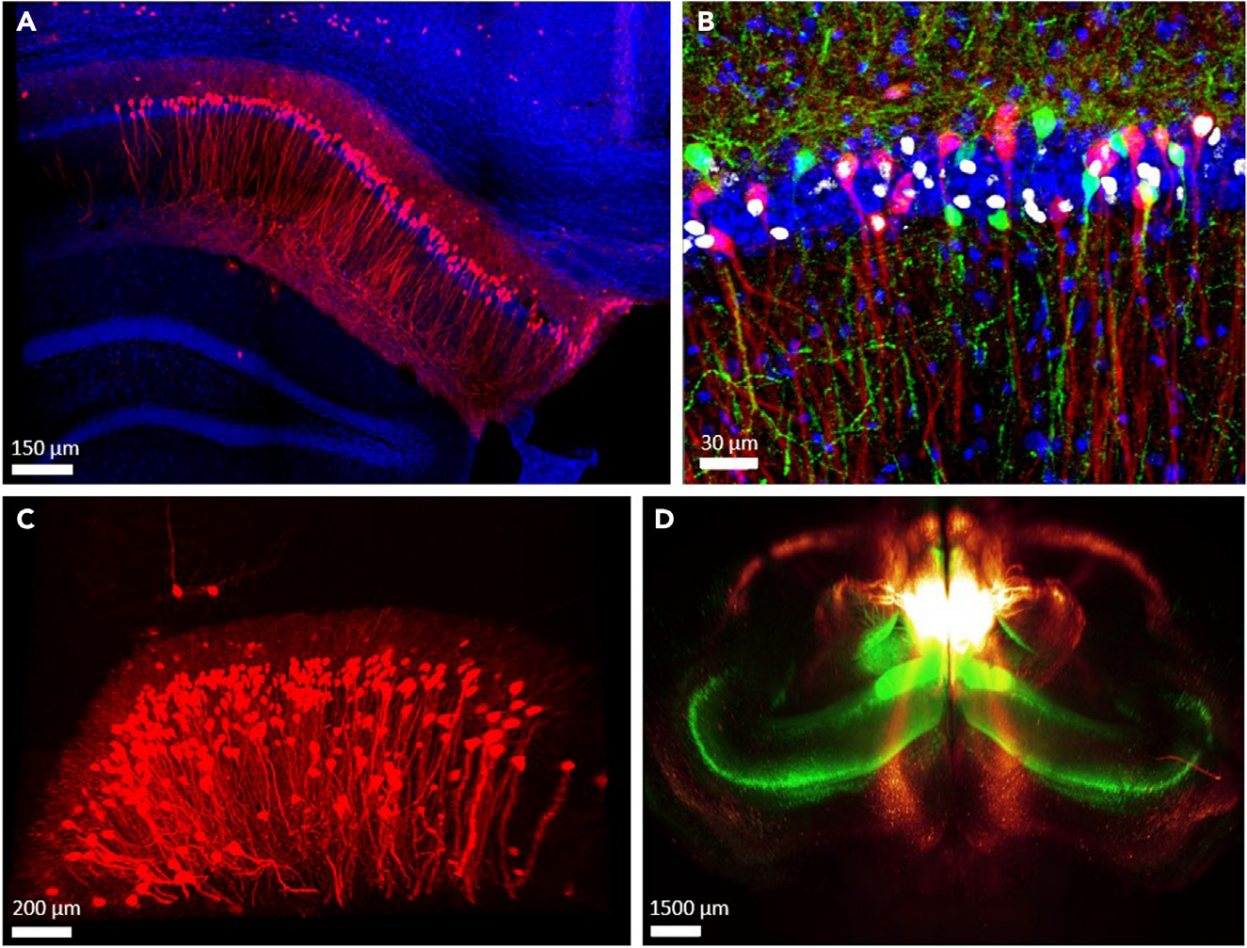
Examples of brain images using different methods (A) Thin slice of the CA1 section of Ai14 reporter mice hippocampus infected with the AAV5-c-Fos::Cre^ER^ vector. Each red cell (TdTomato^+^) expressed c-Fos when the 4-OHT was introduced. When the drug administration is timed to the behavioral paradigm, those cells can be referred to as the engram cells of that behavioral task. (B) The CA1 section of the hippocampus in Ai14 mouse infected with AAV5-c-Fos::Cre^ER^, all red cells (TdTomato^+^) represent the engram cells of the first time point. All green cells (GFP^+^) are CA1→ACC projecting cells, due to injection of the retrograde vector to the ACC (AAVretro-CaMKII::sGFP), and all white cells represent the engram population of the second time point, after staining against the c-Fos protein (AF 647), adjacent to the memory task. (C) TdTomato^+^ cells in thick sample. Using the CLARITY protocol, a large portion of the dorsal CA1 can be easily imaged under a confocal microscope, to allow 3D information of the chosen engram population. (D) The use of our modified CLARITY protocol allows imaging of an entire brain, at a resolution of single cells. From above, the hippocampus is visible (green channel) along with every cell projecting to the ACC, due to infection with AAVretro-CaMKII::GFP.

We generated this protocol to comprehensively investigate engram populations at two time points. The first part of the protocol explains how to mark two engram groups in the same animal, activated at different time points. Using this double labeling procedure, we wanted to address whether hippocampal cells activated during recent fear memory retrieval are also re-activated during remote memory recall, or if both recent and remote engrams resemble memory formation engram but differ one another. This protocol can be modified for any double labeling investigation of two populations of cells, by simply changing the TG mice, the viral vectors, or the activating drug (e.g., DOX instead of 4-OHT in Tet mice). The second part of the protocol clarifies how to image and analyze thick brain slices using a modified protocol for the CLARITY clearing technique (Figure 6).

## Limitations

This protocol relies on several external factors, that should be addressed and validated before starting, such as the validity of the TG mice and the viral vectors, the recommended injected volume of the viral vector (on our example, 400 nL vector from our own virus facility was enough to infect the entire dorsal CA1, but this volume can vary for vectors from different sources), etc.

The protocol process takes a long period of time (several months): Waiting for the protein encoded by the viral vector to express in the brain cells (⁓3 weeks), the behavioral paradigm which involves remote recall (>month) and the CLARITY process which can take more than a month alone.

Another limitation of the CLARITY protocol is related to the imaging process. Before applying this protocol, one should address the issue of the objective properties. As mentioned in step 13, the chosen objective should have high working distance, which should not come at the expense of the numerical aperture or the magnification. Some commercial companies advertise CLARITY optimized objectives.

## Troubleshooting

### Problem 1

**Figure 7:**
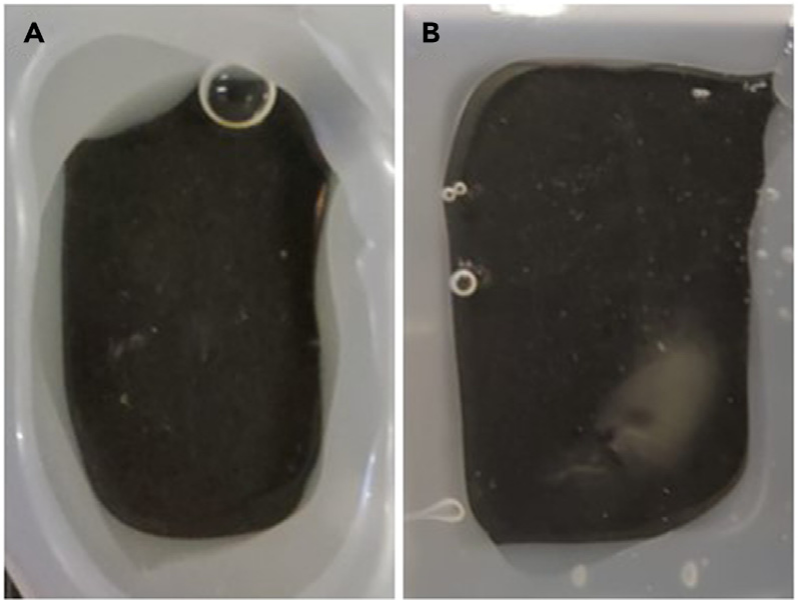
After the chamber is created, aggregates may form in the brain (A) Transparent brain in a chamber. (B) Aggregates appear in the brain, whitening it and harming its structure.

CLARITY protocol is a straightforward procedure, yet it is important to notice that the reagents of each step easily interact with the reagents in the next step, therefore the washing steps are critical, and should not be overlooked. For example, if Triton is not removed sufficiently before immersion into the CSRC refractive index matching solution, it may aggregate, whiten the tissue and harm it (step 12, Figure 7).

### Potential solution

• Avoid interaction of triton with the CSRC by carefully removing it before immersion.
• If aggregates appear, the tissue can be moved back to PBS, and then further back to PBST until the aggregates are expelled. When the tissue is clear again, the procedure can be recommenced starting at step 10.

### Problem 2

Since the final transparency process (step 11) is not part of the clearing process (step 9), it is possible that the tissue will not be transparent enough by the end.

### Potential solution

• In general, the CLARITY protocol steps can be reversed; Remove the tissue back to PBS, then to PBST and lastly back to Clearing Solution 2 for several extra days.
• This back and forth process is not indefinite, as at some point the tissue will lose its rigidity and consequently its structure.

## Resource availability

### Lead contact

Further information and requests for resources and reagents should be directed to and will be fulfilled by the lead contact, Ron Refaeli (ron.refaeli@mail.huji.ac.il).

### Technical contact

Technical questions about this protocol should be directed to the technical contact, Tirzah Kreisel (tirzah.kreisel@mail.huji.ac.il)

### Materials availability

This study did not generate new unique reagents.

### Data and code availability

The published article includes all codes generated or analyzed during this study.
